# Influence of Framework Material and Posterior Implant Angulation in Full-Arch All-on-4 Implant-Supported Prosthesis Stress Concentration

**DOI:** 10.3390/dj10010012

**Published:** 2022-01-14

**Authors:** João Paulo Mendes Tribst, Dayana Campanelli de Morais, Jefferson David Melo de Matos, Guilherme da Rocha Scalzer Lopes, Amanda Maria de Oliveira Dal Piva, Alexandre Luiz Souto Borges, Marco Antonio Bottino, Antonio Lanzotti, Massimo Martorelli, Pietro Ausiello

**Affiliations:** 1 Department of Dental Materials, Academic Centre for Dentistry Amsterdam (ACTA), University of Amsterdam and Vrije Universiteit Amsterdam, 1081 LA Amsterdam, The Netherlands; joao.tribst@gmail.com (J.P.M.T.); amodalpiva@gmail.com (A.M.d.O.D.P.); 2 Department of Dental Materials and Prosthodontics, Institute of Science and Technology, São Paulo State University-UNESP, São José dos Campos 12220-000, Brazil; dayana.morais@ict.unesp.br (D.C.d.M.); jefferson.matos@ict.unesp.br (J.D.M.d.M.); guilherme.lopes@ict.unesp.br (G.d.R.S.L.); alexandre.borges@unesp.br (A.L.S.B.); marco.bottino@unesp.br (M.A.B.); 3 Fraunhofer JL IDEAS, Department of Industrial Engineering, University of Naples Federico II, 80125 Naples, Italy; antonio.lanzotti@unina.it; 4 School of Dentistry, University of Naples Federico II, Via S. Pansini 5, 80131 Naples, Italy; pietausi@unina.it

**Keywords:** dental implants, finite element analysis, polymers, prosthodontics

## Abstract

This study evaluated the influence of distal implants angulation and framework material in the stress concentration of an All-on-4 full-arch prosthesis. A full-arch implant-supported prosthesis 3D model was created with different distal implant angulations and cantilever arms (30° with 10-mm cantilever; 45° with 10-mm cantilever and 45° with 6-mm cantilever) and framework materials (Cobalt–chrome [CoCr alloy], Yttria-stabilized tetragonal zirconia polycrystal [Y-TZP] and polyetheretherketone [PEEK]). Each solid was imported to computer-aided engineering software, and tetrahedral elements formed the mesh. Material properties were assigned to each solid with isotropic and homogeneous behavior. The contacts were considered bonded. A vertical load of 200 N was applied in the distal region of the cantilever arm, and stress was evaluated in Von Misses (σVM) for prosthesis components and the Maximum (σMAX) and Minimum (σMIN) Principal Stresses for the bone. Distal implants angled in 45° with a 10-mm cantilever arm showed the highest stress concentration for all structures with higher stress magnitudes when the PEEK framework was considered. However, distal implants angled in 45° with a 6-mm cantilever arm showed promising mechanical responses with the lowest stress peaks. For the All-on-4 concept, a 45° distal implants angulation is only beneficial if it is possible to reduce the cantilever’s length; otherwise, the use of 30° should be considered. Comparing with PEEK, the YTZP and CoCr concentrated stress in the framework structure, reducing the stress in the prosthetic screw.

## 1. Introduction

Implant prostheses rehabilitation has proven efficacy in the treatment of fully edentulous patients in long-term studies [1,2]. However, the process of bone resorption due to physiological and pathological factors [3] complicates planning for the installation of posterior implants as a prosthetic support [4,5].

The All-on-4 concept [6] consists of the installation of four implants in the medial region of the maxilla or jaw to support fixed or removable prostheses that are immediate, provisional or definitive. Two mesial implants are usually placed perpendicular to the bone crest, and two distal implants are installed inclined, reducing the distal cantilever and allowing replacement of more teeth [7,8,9,10,11]. The predictability and safety of this type of treatment has been already described in the literature [6,12,13,14,15]. During the distal implant installation, different angulation can be used according to surgical planning. However, the literature is not concise regarding the benefits of 30° and 45° angulation for reducing stress concentration [16,17]. It is important to note that the surgeon inserted inclined distal implants to reduce the cantilever length, and this variable should be considered when analyzing prosthetic modality.

In an implant-supported full-arch total prosthesis, it is necessary to use a framework on which artificial teeth are fixed [18]. Among the materials available for the framework, alloys stand out due to their sufficient tensile strength (>300 MPa) and elastic modulus (>80 GPa), which prevent plastic deformations of cantilevers [19]. For that, the CoCr (Cobalt–chrome) alloy is widely used.

With the advent of CAD/CAM technology and increased demand for metal-free prostheses, yttria-stabilized tetragonal zirconia polycrystal (Y-TZP) has been used as an alternative to metal in dental treatments [20]. The use of zirconia allows framework masking and presents a similar survival rate to metal [21,22,23]. Another possibility is the use of polyetheretherketone (PEEK), a high-performance polymer initially applied in orthopedics as a substitute for titanium [24] and currently used in dentistry for the manufacture of implants, frameworks, removable partial dentures and crowns [25,26,27,28,29].

While a metal framework presents an elastic modulus that ranges between 100 and 200 GPa, PEEK presents approximately 4 GPa [26]. Regardless of this difference between the mechanical properties of these materials, it is assumed that PEEK is an alternative material for the manufacture of fixed prosthesis frameworks on implants [28,29]. However, its behavior against the application of stresses should be evaluated.

In this sense, theoretical analysis, e.g., finite element analysis, could be advantageous for investigating the origins of mechanical failure mechanisms around dental implants. This numerical tool can assess stresses and help formulate hypotheses regarding dental implants behavior [30,31,32].

Therefore, the purpose of this study was to evaluate the mechanical behavior in the All-on-4 concept prosthesis with different framework materials (CoCr, YTZP and PEEK) and different distal implants positioning. The null hypothesis was that the use of different framework material and posterior implant position would not affect the prosthesis’ mechanical behavior.

## 2. Materials and Methods

A virtual model of a jaw without maxillofacial abnormalities was exported in DICOM format from the São Paulo State University database. The DICOM file was converted to STL (stereolithography) file in a 3D slicer software. Using the CAD software (Rhinoceros Version 4.0 SR8, McNeel North America, Seattle, WA, USA), a model of an edentulous jaw was created following the main anatomical characteristics of an adult bone: size, shape and absence of pathology [27]. The BioCad method was applied to create a volumetric and solid model [33]. For that, the anatomical surface of the jaw has been delimited by polylines, and the intersection of three or four polylines has been used to form a network surface. The association of juxtaposed and joined surfaces formed the final volume of the 3D model. In sequence, external hexagon implants (10 × 4.1 mm) were created with the external thread diameter being established according to the dimensions provided by the manufacturer (Conexão Sistemas de Prótese, Arujá, Brazil) [18]. The platform had a diameter of 4.1 mm, and the external hexagon presented 0.7 mm of height. The minimum distance between the implants was 4 mm [18,20].

Three different models were created according to distal implant positioning in relation with the implant’s long-axis: 30° with a 10-mm cantilever (30°/10 mm), 45° with a 10-mm cantilever (45°/10 mm) or 45° with a 6-mm cantilever (45°/6 mm).

**Figure 1:**
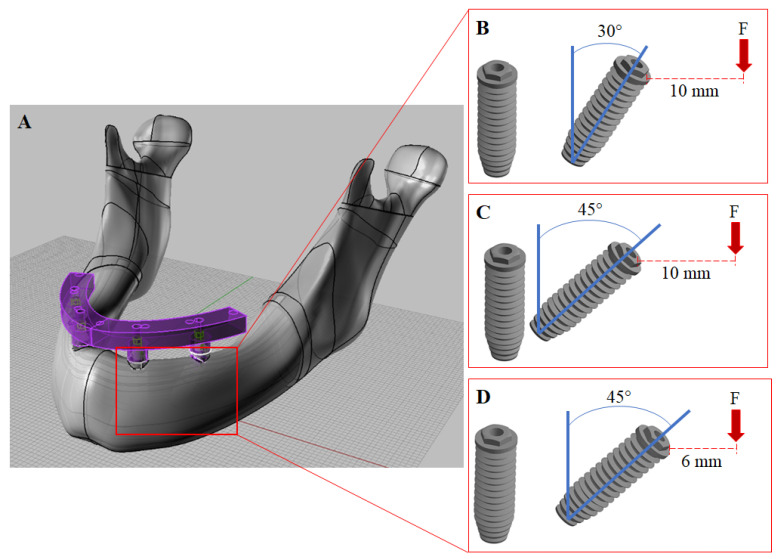
(**A**) Three-dimensional modeling of an All-on-4 full arch prosthesis in perspective view. (**B**) Distal implants with 30° angulation and a 10-mm cantilever. (**C**) Distal implants with 45° angulation and a 10-mm cantilever arm. (**D**) Distal implants with 45° angulation and a 6-mm cantilever. Read arrows (F) represent where the 200 N load was applied.

In this study, the 30° angled implant was considered as a starting point for the All-on-4 technique. Normally, the an All-on-4 concept is performed with two posterior inclined implants. However, implant inclination should be carefully considered during the treatment plan, because it is possible to incline the implant apex or the implant platform to achieve 45° of inclination. The first situation (apex) will keep the cantilever arm similar to the starting point (30°), increasing only the angle of insertion, while the second situation (platform) can reduce the cantilever arm at 40%. For all simulated models, the framework bar that received the load presented the same extension (In Figure 1, the load region in F is similar regardless the model). For that reason, we did not consider 30° with the cantilever of 6 mm, as it would represent a reduction in the extension of the bar and, therefore, a different quantity in the number of teeth.

The framework presented a height of 6 mm from the bone surface and the lever arm following the implant platform position. The screw access holes were created to simulate a clinical situation. Finally, at the end of the framework, a 2-mm diameter delimitation was created to standardize load application [29,33]. The 3D prosthetic screw was modeled for each implant. The geometry designs are presented in Figure 1.

Each solid geometry was imported to the finite element software (ANSYS 17.2, ANSYS Inc., Houston, TX, USA) in STEP format. A 3D mesh was generated, and tetrahedral elements were used. A convergence test of 10% determined the total number of elements and nodes for each model (Table 1).

Young’s modulus and Poisson ratio of each material were assigned to each solid component with isotropic and homogeneous behavior (Table 2).

The jaw model was fixed in all directions on the bottom surfaces, and the contacts were considered bonded. On the delimited distal area (2 mm), a load of 200 N was applied normal to the surface [29,33] (Figure 1). Results were selected in von Mises stress for the framework, implants and prosthetic screw. The maximum (tensile) and minimum (compression) principal stresses criteria were evaluated for bone tissue.

## 3. Results

**Figure 2:**
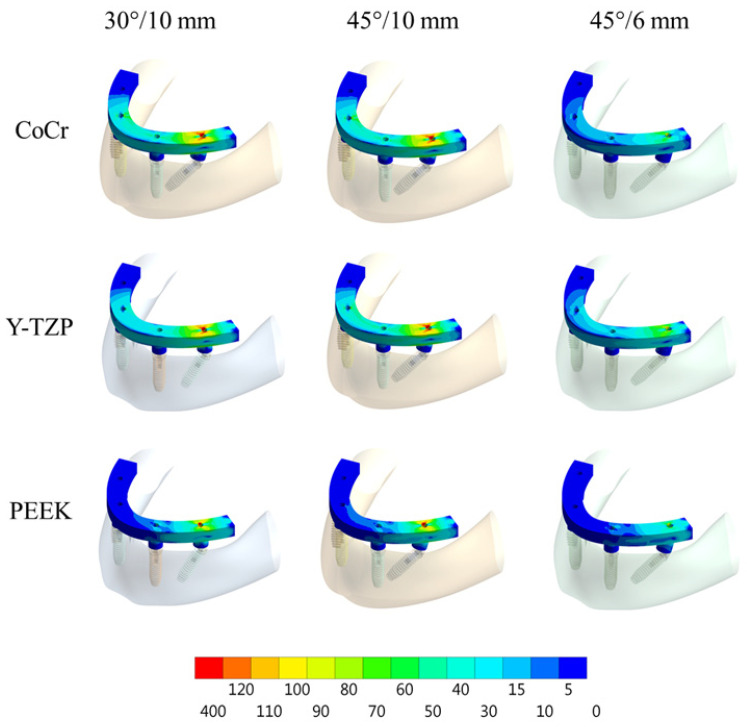
Von Mises stress in the prosthetic framework according to framework material (CoCr, Y-TZP and PEEK) and distal implant position (30° and 10-mm cantilever, 45° and 10-mm cantilever and 45° and 6-mm cantilever).

**Figure 3:**
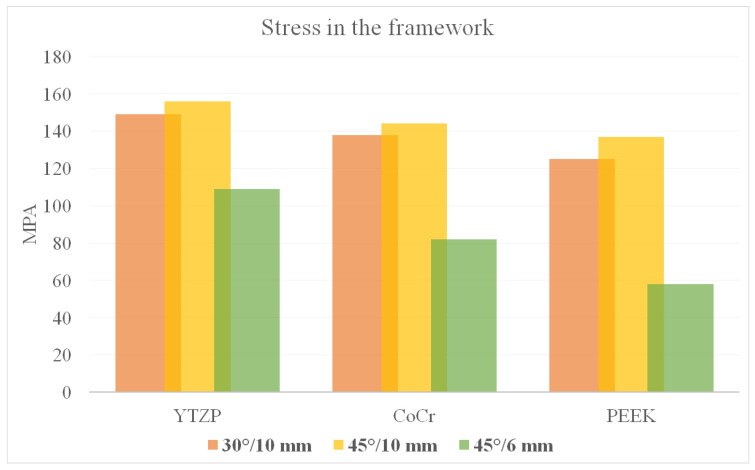
Bar graph with Von Mises stress peaks in the prosthesis’ framework according to material and distal implants position.

**Figure 4:**
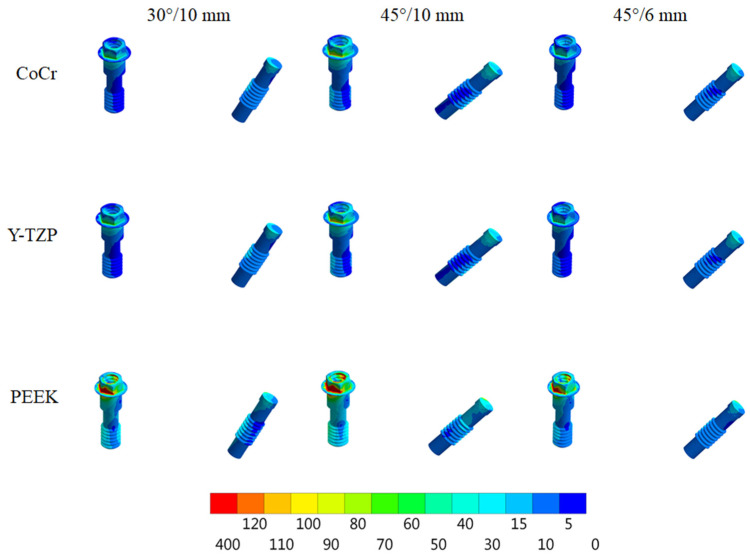
Von Mises stress in the prosthetic screws (anterior and posterior) according to framework material (CoCr, Y-TZP and PEEK) and distal implant position (30° and 10-mm cantilever, 45° and 10-mm cantilever and 45° and 6-mm cantilever).

**Figure 5:**
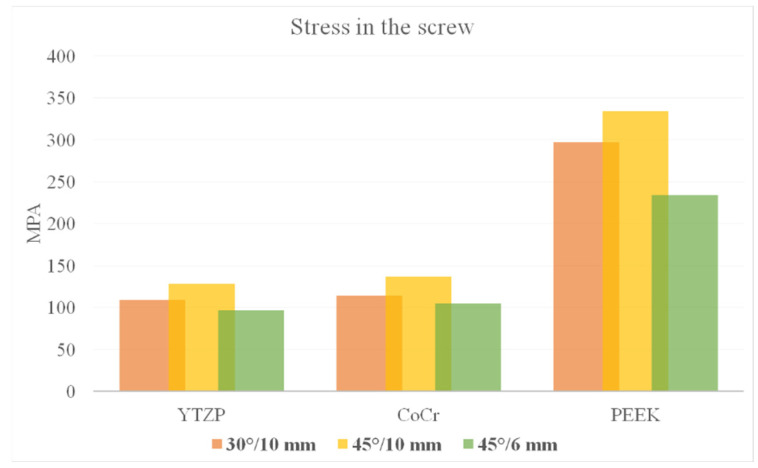
Bar graph with Von Mises stress peaks in the prosthetic screw according to material and distal implant position.

**Figure 6:**
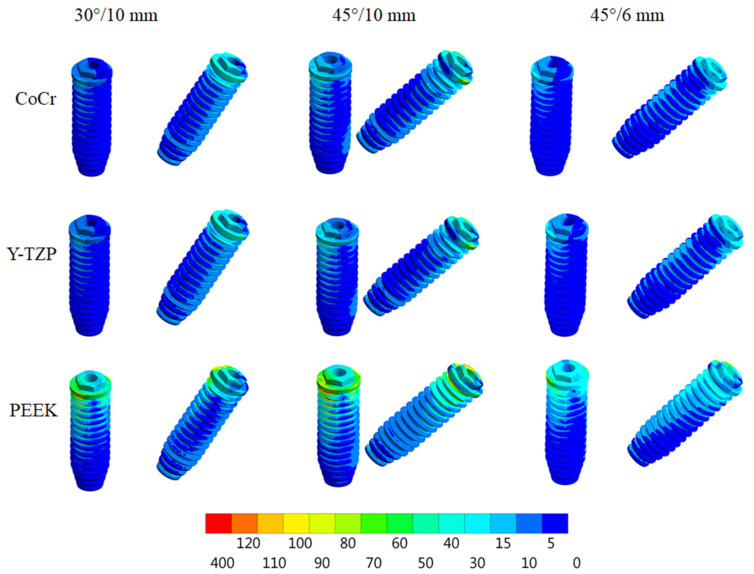
Von Mises stress in the prosthetic implants (anterior and posterior) according to framework material (CoCr, Y-TZP and PEEK) and distal implant position (30° and 10-mm cantilever, 45° and 10-mm cantilever and 45° and 6-mm cantilever).

**Figure 7:**
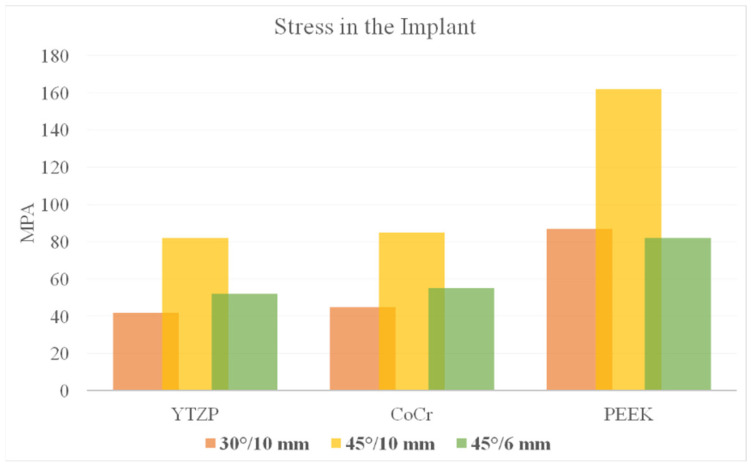
Bar graph with Von Mises stress peaks in the implant according to material and distal implant position.

According to Von Mises stress results in the framework, the lower the material elastic modulus, the lower the stress concentration. In addition, the 45°/6 mm position showed the lowest stress peak values (Figure 2 and Figure 3). The opposite was observed for the prosthetic screw (Figure 4 and Figure 5) and implants (Figure 6 and Figure 7): The lower the framework elastic modulus, the higher the stress concentration. Moreover, the same implant position (45°/6 mm) showed highest peak values in these structures. The use of PEEK framework associated with 45°/10 mm design showed the highest stress magnitude in the screw and in the implants. Regardless the framework material and design, the highest stress concentration was calculated in the first thread for the prosthetic screw in the mesial implants. The maximum principal and minimum principal stresses peaks were plotted in the bar graphs. Stress peaks have been collected using the auto-tool max probe in mechanical APDL software after processing the results. Regardless of stress magnitude, the stress distribution trend was similar between the models, with the highest stress in the prosthetic screw head and implant platform.

**Figure 8:**
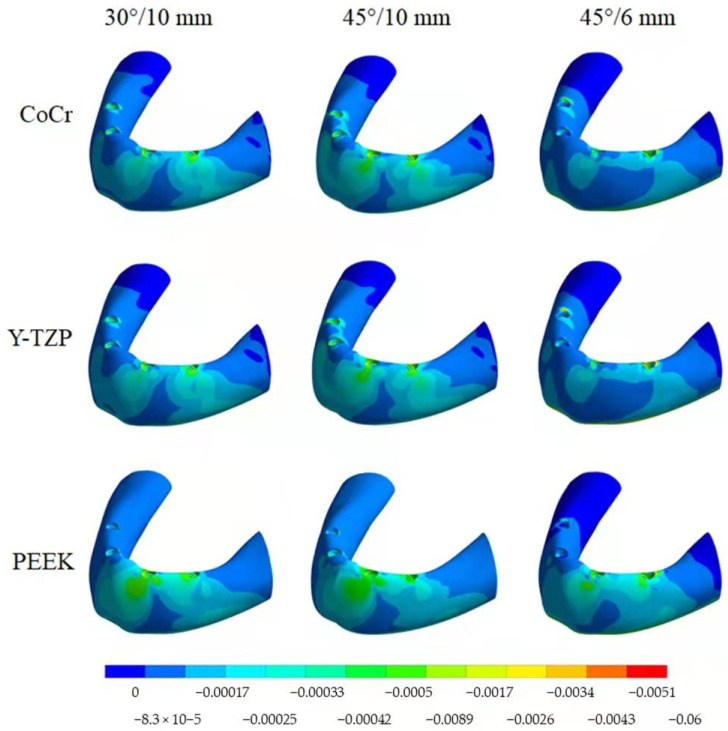
Tensile stress (σMAX) in the bone according to material (CoCr, Y-TZP and PEEK) and distal implant position (30° and 10-mm cantilever, 45° and 10-mm cantilever and 45° and 6-mm cantilever).

**Figure 9:**
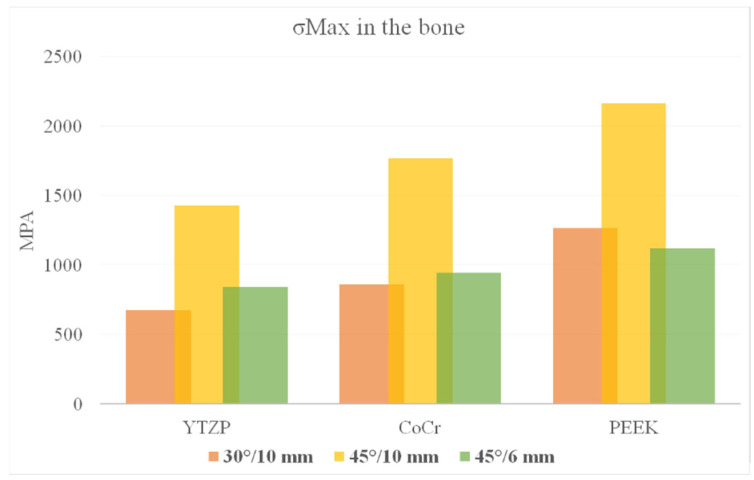
Bar graph with Tensile (σMAX) stress peaks in the bone according to material and distal implant position.

**Figure 10:**
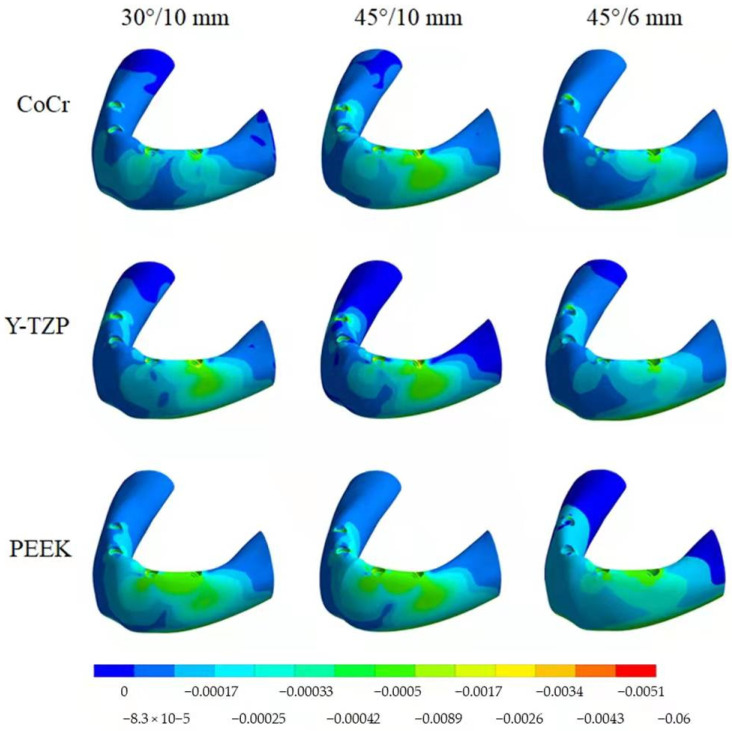
Compressive stress (σMIN) in the bone according to material (CoCr, Y-TZP and PEEK) and distal implant position (30° and 10-mm cantilever, 45° and 10-mm cantilever and 45° and 6-mm cantilever).

**Figure 11:**
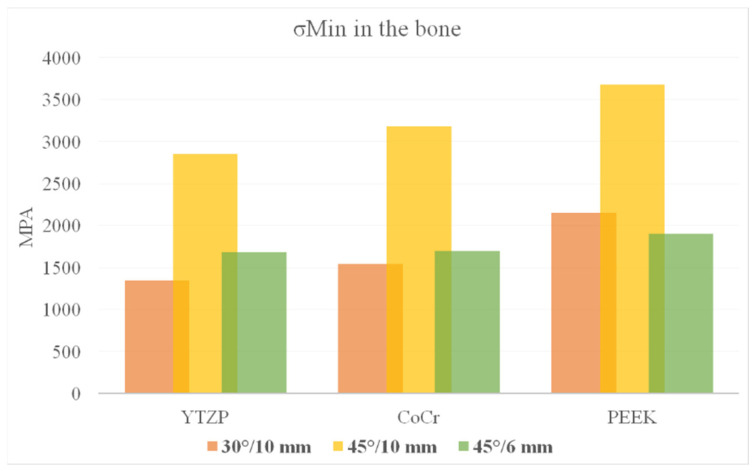
Bar graph with Compressive (σMIN) stress peaks in the bone according to material and distal implant position.

The highest σMAX magnitude was calculated in the mesial implants in the cervical buccal region (Figure 8 and Figure 9), while σMIN peaks were concentrated in the cervical area of the distal implant (Figure 10 and Figure 11). Regarding bone stress, the framework in PEEK resulted in lower σMAX and higher the σMIN in bones. The 45°/10 mm position showed the highest concentration for both criteria, while the 45°/6 mm position resulted in the lowest stress concentration. The 30°/10 mm position showed intermediate behavior, suggesting that higher implant angulation should only be used if the cantilever can be reduced with it.

## 4. Discussion

The present study evaluated the stress distribution in All-on-4 prosthesis regarding framework material and posterior implant angulation. The results showed that both factors affected the mechanical response during a vertical load simulation. Thus, the hypothesis was rejected.

According to the literature [33,34], the vertical load in the molar region can vary from 150 to 800 N, and there is a reduction of 30% to 40% in terms of the amount of load when applied unilaterally compared to bilateral applications [35]. In this study, load was applied with 200 N reported as the mean value of occlusal force in the posterior region in implant-supported prosthesis [29,36].

A previous report [37] performed a 3D-finite element model of an edentulous jaw, and a vertical load of 200N was applied at the cantilever similar to the present study. The authors affirmed that the framework material (metal or acrylic resin) did not play an important role in reducing peri-implant bone strain and that a reduced cantilever improved the mechanical response. The present study is in agreement with the benefits provided by a short cantilever arm; however, the results presented herein suggest that a flexible framework seems to be able to increase peri-implant stress (Figure 2 and Figure 3).

Regarding the framework elastic modulus, a previous study [38] found that PEEK framework showed highest stress peaks in comparison with more rigid materials for the prosthetic screw. The present study corroborates with this finding (Figure 4 and Figure 5) and agrees that further studies are necessary for evaluating screw performance. A prospective cohort clinical study with 37 patients analyzed the short-term outcome of full-arch implant-supported fixed hybrid PEEK prostheses in the All-on-4 concept [39]. The authors suggest that PEEK resin prostheses for full-arch rehabilitation may represent a valid treatment option. The authors also reported that five prostheses presented prosthetic screw loosening. This can be explained with the increased stress concentration in the screw, as showed in the present study’s results.

Higher stress magnitude was observed in the mesial screws, agreeing with other studies [10,17,18,20] and showing similarity to the result found in the bone where tensile stress was higher in the region of the mesial implants. This can be justified because it is a more distant position of the cantilever that undergoes movement during the application of load in this region, functioning as a lever system.

In another clinical study [40], the authors performed a three-year prospective evaluation from PEEK framework with the All-on-4 concept. The authors suggest that the proposed rehabilitation solution is a legitimate treatment option. From thirty-seven patients, three patients presented screw loosening and one patient presented peri-implant pathology solved with surgery. These complications can be associated with the use of a flexible framework able to bend during chewing and able to stress the screw and bone, as shown in the present study. However, this is only one hypothesis from what could occur.

The prosthesis was designed by varying the length of the cantilever, since the 45° angulation of the distal implants would change its extension. Indeed, the 45° implant and reduced cantilever (45°/6 mm) showed less stress magnitude (Figure 6 and Figure 7) corroborating with previous studies that verified a direct relationship between the length of the cantilever and stress concentration [7,8,9,10,11]. However, if the cantilever length was not reduced and implant angulation increases (45°/10 mm), the stress concentration will increase in comparison with the 30° angulation group.

With photo-elastic strain analysis, a previous study [41] found that metal and zirconia showed strain patterns lower than PEEK with the All-on-4 concept. The authors justify these results by asserting that an increased elastic modulus of the framework reduced the stresses transmitted to the implants and bone. The present study is in agreement with this statement since a similar mechanical behavior for the implants and bone (Figure 8, Figure 9, Figure 10 and Figure 11) was observed with similar materials and model design.

Bone behavior was evaluated in tensile (σMAX) and compression (σMIN) stresses [42,43,44]. In compression, the highest magnitude was found near the cervical area of the distal implant near of the load application site (distal cantilever region) (Figure 10). Therefore, the distal implant suffered more compression, and the mesial implant showed the highest tensile stress (Figure 8), a behavior that is in agreement with the results of previous studies [10,43,44,45]. In this case, the framework material that transferred more stress to the bone in both compression and tensile stress was PEEK (Figure 9 and Figure 11).

The influence of posterior implant angulations has already been reported in the literature [46]. By using 3D finite element analysis (3D-FEA), a previous study affirmed that stress increased when implants increased in angulation; however, when used with a short cantilever, inclined implants decreased stress on peri-implant cortical bone. The present study corroborates with these results, showing the same mechanical behavior for the 45° with the 6-mm model.

The literature presents a higher survival rate (90%) for implant-supported full-arch prosthesis with longer cantilevers (7–12 mm) after 15 years of clinical follow-up [2]. Thus, the use of cantilever is a clinical option that can be used in some cases of rehabilitation, resulting in clinical success in the long term but at the same time depends on correct planning and biomechanics understating.

Despite dental PEEK presenting a wide clinical indication, such as fixed dental prostheses [47], removable partial dentures [48] and implant-supported frameworks [49], its use should be applied with caution due to the increase in stress contraction in prone-to-failure regions, e.g., prosthetic screws. In addition, further studies can be carried out evaluating the interaction between resinous cement and polymeric structures for cement-retained frameworks, focusing on polymerization shrinkage Kinects [50,51].

A previous study aimed to analyze the effects of different materials used for framework fabrication on the “All-on-4” implant system by using the finite element method. The authors considered three framework materials (Titanium, Zirconia and PEEK) in the study for the evaluation of stresses in peri-implant regions [52]. According to their simulation, the stress distribution pattern at the implant–bone interface was influenced by the framework material used while a zirconia framework showed minimal stress magnitude. In addition, the length of cantilever increased stress concentrations regardless the framework materials [52]. Therefore, the present study corroborates with that result by showing an increased stress trend with higher cantilever arm (10 mm) in comparison with reduced one (6 mm).

According to the literature [53], increasing the inclination in posterior implants resulted in a reduction in cantilever length and maximum stress decline in both cancellous and cortical bone. The effect of cantilever length seems to be a dominant factor for the stress magnitude [53]. Therefore, the present study corroborates with this statement by showing that reduced cantilever length using a higher inclination for the implant’s placement can be beneficial. In addition, the cantilever length should be measured from the most posterior implant platform, confirming that the inclination of the implant platform was done instead of the implant apex.

Applying the current advances in dental implants associated with the All-on-4 treatment concept generally reduces treatment time, risk of morbidity and other possible risks in the edentulous patient [54]. In order to prove this, a previous 3D-FEA study showed that the use of implant-supported ceramic prosthesis as an antagonist to another implant-supported full arch prosthesis increases the amount of stresses transmitted to the bone and that there is no difference in the amount of stresses when comparing natural tooth to tooth-supported ceramic restorations as an antagonist [54]. The authors affirmed that the model design followed the classic All-on-4 configuration by tilting posterior implants to a 30° angle, but changing the angle or the configuration of the implants could result in different results from a biomechanical standpoint [54]. The present study demonstrates that this hypothesis is correct; indeed, posterior implant inclination can affect the entire system mechanical responses.

The use of in vitro investigations also can be used to describe mechanical responses. A previous investigation [55] applied linear strain gauges to measure the strain during compressive loading in full-arch rehabilitations. According to the results, the amount of peri-implant strain in All-on-4 implant-supported prosthesis depends on the selection of framework material, cantilever length and the occlusal forces from the opposing arch. In addition, when mandibular All-on-4 implant-supported prostheses are placed opposite to parafunctional forces, the presence of the cantilever might result in peri-implant strain generation beyond the physiological limits of bones, irrespective of the type of framework material [55]. For that reason, reducing the cantilever size can be a promising approach during the treatment plan.

The present study considered only unilateral loading conditions, showing asymmetric stress distribution between model extensions. The use of posterior loading with bilateral occlusion could modify the results and stress magnitude; however, the difference in the groups could possibly be proportional to the present condition, as previously demonstrated [56,57]. Another variable that was not evaluated in the present study is the different implant connections [58,59,60,61]. The use of an external hexagon implant system can present a different mechanical response from internal connection systems, e.g., internal hexagon and Morse-taper, affecting peri-implant tissue stability and prognosis [58,59,60,61]. Therefore, new stress analyses should be performed by considering these variables during the modelling step, which aims to corroborate the present study results.

The method of 3D-FEA has been widely used to simulate clinical situations and provide scientific data. However, it is relevant to mention that this study did not simulate the dynamic medium that is the human mouth. Thus, humidity, chewing, pH and temperature variations and other bone conditions should be evaluated in further investigations to corroborate with the present findings.

## 5. Conclusions

For the All-on-4 technique, an angulation of 45° for distal implants is only beneficial if it is possible to reduce the cantilever’s length. Otherwise, 30° should be considered as the first choice. In addition, stiffer framework materials concentrated more stress in its structure, reducing prosthetic screw stress magnitude.

## Figures and Tables

**Table 1:** Number of nodes and elements used in the analysis for each model.

Inclination of Distal Implants (°)	Distal Cantilever (mm)	Nodes	Elements
30	10	1.133.298	645.591
45	10	1.037.324	625.014
45	6	1.084.148	597.018

**Table 2:** Mechanical properties of materials used in finite element analysis.

Structure	Elastic Modulus (GPa)	Poisson Ratio
Cancellous bone	1.37	0.3
Cortical bone	13.7	0.3
CoCr	210	0.3
Y-TZP	200	0.3
PEEK	4	0.3

## Data Availability

Data available upon request.
